# Subjective and objective evaluation of 10–30% dose reduced coronary artery phantom scans reconstructed with Forward projected model-based Iterative Reconstruction SoluTion (FIRST)

**DOI:** 10.1016/j.dib.2016.11.084

**Published:** 2016-12-03

**Authors:** Eriko Maeda, Nobuo Tomizawa, Shigeaki Kanno, Koichiro Yasaka, Takatoshi Kubo, Kenji Ino, Rumiko Torigoe, Kuni Ohtomo

**Affiliations:** a Department of Radiology, Graduate School of Medicine, University of Tokyo, 7-3-1 Hongo, Bunkyo-ku, Tokyo 113-8655, Japan; b Department of Radiology, New Tokyo Hospital, 1271 Wanagaya, Matsudo-city, Chiba 270-2232, Japan; c Imaging Center, The University of Tokyo Hospital, 7-3-1 Hongo, Bunkyo-ku, Tokyo 113-8655, Japan; d Toshiba Medical Systems Corporation, Tokyo Metropolitan Regional Office, 1-6, Tsukuda 2-Chome, Chuo-ku, Tokyo 104-0051, Japan

**Keywords:** Coronary CT angiography, Image quality, Iterative reconstruction, Radiation dose, 320-row CT

## Abstract

The data presented in this articles are related to the research article entitled “The feasibility of Forward-projected model-based Iterative Reconstruction SoluTion (FIRST) for coronary 320-row computed tomography angiography: a pilot study” (E. Maeda, N. Tomizawa, S. Kanno, K. Yasaka, T. Kubo, K. Ino, R. Torigoe, K. Ohtomo, 2016) [1]. This article describes subjective and objective evaluations of 2 mm–4 mm coronary artery phantom scanned with 100% dose and reconstructed with hybrid iterative reconstruction, and 90%, 80% and 70% dose reconstructed with full iterative reconstruction.

**Specifications Table**

**Table:** 

Subject area	Radiology
More specific subject area	Effect dose reduction on image quality in CT images reconstructed with full iterative reconstruction.
Type of data	Table, image, text file
How data was acquired	Coronary phantoms were scanned with computed tomography at various doses.
	100% dose images were reconstructed with hybrid iterative reconstruction, while other dose images were reconstructed with full iterative reconstruction.
	Subjective and objective image quality of each images were assessed.
Data format	Raw, Analyzed
Experimental factors	The subjective and objective image quality of coronary artery phantom scanned with various dose were evaluated by 3 radiologists.
Experimental features	The relationship between the degree of dose reduction and image quality was determined for images reconstructed using Forward-projected model-based Iterative Reconstruction SoluTion (FIRST).
Data source location	Hongo, Bunkyo-ku, Tokyo 35^°^ 42^′^ 45.64^″^ N; 139^°^ 45^′^ 43.16^″^ E
Data accessibility	The data are available with this article

**Value of the data**

• The data is the description of the effect of FIRST reconstruction for −10 to −30% dose reduced CT images.
• Researchers can compare the radiological appearance of coronary phantoms in different diameters (4 mm, 3 mm, and 2 mm) in various doses reconstructed with FIRST.
• This data allows other researchers to determine how much tube current can be lowered when using full iterative reconstruction in clinical settings.

## 1 Data

**Fig. 1:**
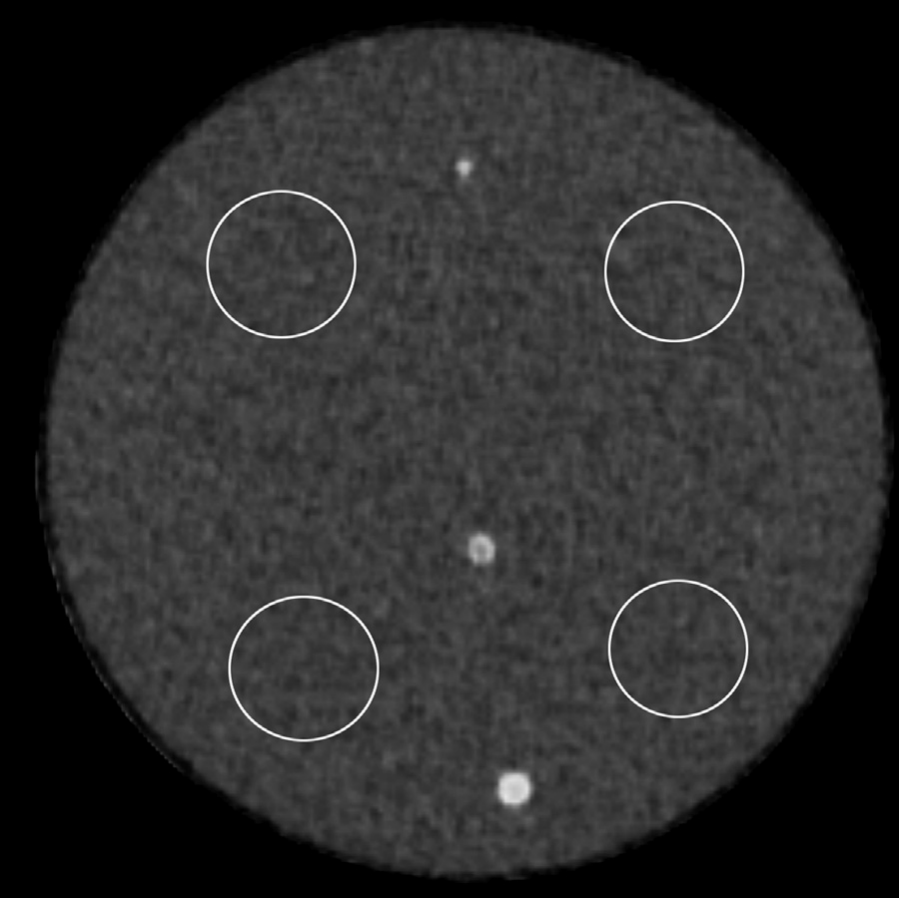
Unenhanced CT image of coronary artery phantom and region-of-interests placed on right upper, left upper, right lower and left lower quadrants.

The data was acquired as a preliminary study for the research article entitled “The feasibility of Forward-projected model-based Iterative Reconstruction SoluTion (FIRST) for coronary 320-row computed tomography angiography: a pilot study " [1]. The dataset of this article provides objective and subjective evaluations of 2 mm–4 mm coronary artery phantom scanned with 10%, 20% and 30% reduced dose from AIDR3D group and reconstructed with FIRST with 3 to 6 boards on each sides of the phantom. Fig. 1 represents the CT image of the phantom and 4 ROIs used for objective evaluation. Table 1, Table 2, Table 3, Table 4 show objective image quality in standard deviation, and subjective scores for each diameter of coronary artery phantom. Table 1 represents data for 3 boards (representing patients weighing 40–49 kg), Table 2 represents 4 boards (50–59 kg), Table 3 represents 5 boards (60–69 kg), and Table 4 represents 6 boards (70–79 kg).

## 2 Experimental design, materials and methods

### 2.1 The phantom

Three polyethylene tubes 4 mm, 3 mm, and 2 mm in diameters were filled with contrast material (Iopamiron 370, 370 mg/mL; Bayer, Osaka, Japan) and was perpendicularly fixed in a polypropylene cylinder. The cylinder was filled with water and was sealed with polypropylene. Three to six acrylate boards were placed on each sides of the coronary artery phantom: 3 boards represented patients weighing 40–49 kg, 4 boards represented 50–59 kg, 5 boards represented 60–69 kg, and 6 boards represented 70–79 kg. This set of phantom was used in the previous study [2].

### 2.2 Image acquisition

All scans were performed using second-generation 320-row CT (Aquilion ONE ViSION edition; Toshiba, Tochigi, Japan). Prospective electrocardiogram-triggered axial scans of all patients were performed using the following parameters: detector configuration, 320×0.5 mm; tube potential, 120 kVp; scan range 60 mm; scan mode 75% Target CTA gated to synthetic ECG signal emitted by the electrocardiograph. The reconstructed slice thickness was 0.50 mm, and the increment was 0.5 mm. Images were reconstructed using a medium soft tissue kernel (FC04). For each number of acrylate boards, initial tube current was determined as the standard deviation of AIDR3D (Adaptive Iterative Dose Reduction with sureexposure3D settings) in enhanced strong mode reconstructed image being 18. The initial tube current scan was reconstructed using AIDR3D and filtered back projection (FBP). Then 10%, 20% and 30% reduced tube current was employed and was reconstructed using FIRST (in Cardiac strong mode) and FBP. The middle slice of the volume (i.e. at 30 mm) was used for further evaluation.

### 2.3 Objective evaluation

Four regions of interests (ROIs) were placed in the right upper, right lower, left upper and left lower quadrants of the images (Fig. 1). The SD of each ROIs were averaged to calculate the SD of the image. The positions of the ROIs were copied to other images, so the ROIs were placed on exactly the same places for all images.

### 2.4 Subjective evaluation

For each image, sharpness of 4 mm, 3 mm and 2 mm coronary artery phantoms were evaluated using 5-point scale (5: excellent, 1: poor) by 3 radiologists with 14, 9, and 7 years’ experiences in cardiovascular imaging. The scores of the three radiologists were averaged to derive the final subjective score for the image.

## Figures and Tables

**Table 1:** Data for 3 boards on each sides of the phantom.

Reconstruction	Initial dose	−10% dose	−20% dose	−30% dose
	eAIDR	FIRST	FIRST	FIRST
Tube current (mA)	150	140	120	100
Standard deviation (Hounsfield units)	16.25	16.69	17.05	16.65
Subjective scores				
4 mm phantom	3	4	4	4
3 mm phantom	3	3	3	2
2 mm phantom	3	4	3	4

**Table 2:** Data for 4 boards on each sides of the phantom.

Reconstruction	Initial dose	−10% dose	−20% dose	−30% dose
	eAIDR	FIRST	FIRST	FIRST
Tube current (mA)	200	180	160	140
Standard deviation (Hounsfield units)	16.67	16.44	16.37	16.62
Subjective scores				
4 mm phantom	3	4	4	5
3 mm phantom	3	4	4	4
2 mm phantom	3	4	4	4

**Table 3:** Data for 5 boards on each sides of the phantom.

Reconstruction	Initial dose	−10% dose	−20% dose	−30% dose
	eAIDR	FIRST	FIRST	FIRST
Tube current (mA)	250	230	200	180
Standard deviation (Hounsfield units)	18.98	16	16.87	16.65
Subjective scores				
4 mm phantom	3	4	3	3
3 mm phantom	3	3	2	3
2 mm phantom	3	4	3	2

**Table 4:** Data for 6 boards on each sides of the phantom.

Reconstruction	Initial dose	−10% dose	−20% dose	−30% dose
	eAIDR	FIRST	FIRST	FIRST
Tube current (mA)	350	320	280	250
Standard deviation (Hounsfield units)	16.82	16.33	17.28	16.48
Subjective scores				
4 mm phantom	3	5	4	4
3 mm phantom	3	2	3	3
2 mm phantom	3	3	2	2

## References

[1] E. Maeda, N. Tomizawa, S. Kanno, K. Yasaka, T. Kubo, K. Ino, R. Torigoe, K. Ohtomo. The feasibility of Forward-projected model-based Iterative Reconstruction SoluTion (FIRST) for coronary 320-row computed tomography angiography: a pilot study, J Cardiovasc Comput Tomogr. 2016 Nov 9. pii: S1934-5925(16)30273-8. http://dx.doi.org/10.1016/j.jcct.2016.11.002. [Epub ahead of print] 10.1016/j.jcct.2016.11.002 27894902

[2] Tomizawa N., Nojo T., Akahane M., Torigoe R., Kiryu S., Ohtomo K. (2012). Adaptive iterative dose reduction in coronary CT angiography using 320-row CT: assessment of radiation dose reduction and image quality. J. Cardiovasc. Comput. Tomogr..

